# Rural–urban differences in suicide attempts and mortality among young people in northern Sweden, 1998–2017: A register-based study

**DOI:** 10.1177/1403494820939018

**Published:** 2020-07-17

**Authors:** Miguel San Sebastián, Anette Edin-Liljegren, Frida Jonsson

**Affiliations:** 1 Department of Epidemiology and Global Health, Umeå University, Umeå, Sweden; 2 Centre for Rural Medicine, Region Västerbotten, Storuman, Sweden; 3 Department of Nursing, Umeå University, Umeå, Sweden; 4 Division of Innovative Care Research, Karolinska Institutet, Stockholm, Sweden

**Keywords:** Suicide attempts, suicide mortality, rural, youth, northern Sweden

## Abstract

*Aim:* The aim of this study was to assess trends in suicide attempts and mortality between 1998 and 2017 by residential area and gender among northern Swedish youths. *Methods:* Events of suicide attempts and deaths for each municipality in the four counties of the northern Swedish region, stratified by gender, were retrieved for each year from 1998 to 2017. All cases identified within the registers with the diagnostic codes X60–X84 or Y10–Y34 (International Classification of Diseases, 10th revision) for individuals aged 15–24 years were included. Place of residence at municipal level was categorized into three groups: rural – municipalities with a population of <10,000 inhabitants; semi-rural – those between 10,000 and 50,000; and urban – those with >50,000 inhabitants. *Results:* The rates of attempted suicides in the northern region were higher in both men and women than in Sweden, while the rates of suicide deaths were slightly higher in young men but similar in young women compared to the national averages. Overall, the risk of suicide attempts was higher in semi-rural municipalities compared to urban ones, particularly among women. A significantly higher risk of mortality was also observed for men and women in semi-rural municipalities, but only in the period 2010–2013. ***Conclusions:* The high rates of attempted and completed suicides among youth in northern Sweden and the higher risks in rural and semi-rural municipalities need close attention. The implementation of suicide prevention programs, especially in rural and semi-rural municipalities, should be intensified.**

## Background

Suicide comprises a serious and sensitive public health problem that is not only tragic in its own right following the loss of a specific life, but also in consideration of the families and communities that are left behind [1]. In the Scandinavian countries, death by suicide has followed a decreasing trend in the total population over the last 30 years, while upward or stable rates have been observed among young people in certain contexts [2]. This pattern has run in parallel to the growing number of youth aged between 15 and 24 years in the region that have tried to take their own life [2–4].

The National Centre for Suicide Research and Prevention of Mental Ill-Health in Sweden has maintained a register of all attempted and completed suicides in the country by age and gender since 1980 [5]. For groups over the age of 25, suicide rates decreased until the year 2000 and stabilized thereafter. While the overall rates are lower compared to other age groups, an increase of almost one percent per year has simultaneously occurred for youth suicide deaths since 1994; in 2017, the rate in young men and women was 17.6 and 7.5 per 100,000 population, respectively [3]. In partial contrast to this trend, suicide attempts in Sweden have followed a different pattern. While the highest rates have been seen among youth, a downward trend has been observed since 2008, with the latest 2016 figures showing a rate per 100,000 population of 106.2 in men and 252.3 in women [4].

In the international literature, suicide deaths in the general population typically show a rural–urban disparity to the disadvantage of rural areas [6–10]. This pattern has also been observed among young people, where rural youth – especially young men – appear to take their own life to a greater extent than their urban counterparts [11–13]. To explain the inequalities, a range of interconnected factors at different levels have been proposed. Specifically, beyond traditional risks like depression and substance abuse, aspects of rural settings such as geographical isolation, limited access to services, socioeconomic decline, masculinity norms and mental health stigma may contribute to the rural–urban suicide gap [14,15].

Knowledge of the patterning of youth suicidal behaviours across regions and rural–urban settings is currently limited in Sweden. To fill this gap and provide policy-relevant information that could guide the development of locally appropriate interventions, the aim of this study was to assess trends in suicide attempts and mortality between 1998 and 2017 by gender and rural–urban areas among northern Swedish youths.

## Methods

### Setting

Northern Sweden occupies approximately 60% of the Swedish land area but includes only around 12% of the total population. The region is divided into four counties (Norrbotten, Västerbotten, Västernorrland and Jämtland) and 44 municipalities, of which 66% are rural. This area is home to the Sámi population, which comprises around 20,000–40,000 individuals [16], as well as an increasing number of international migrants, of which many are unaccompanied children and youth. The area has had a historical dependence on mining and forestry industries, and a nationally high prevalence of cardiovascular disease. As such, the social and public health landscape, as well as the prerequisites for equity in health, differ from the regions of the more populated south.

Sweden implemented a programme with nine strategic areas of action for reducing the number of suicides in 2008, which is coordinated by the Public Health Agency of Sweden at national level. A recent study concluded that the National Suicide Prevention Program was effective, but dependent on age and sex [17,18].

### Data source

Events of youth attempted and completed suicides for each municipality in the four counties of the northern Swedish region, stratified by gender, were retrieved from the National Board of Health and Welfare’s inpatient and death registers, respectively, for each year from 1998 to 2017. Specifically, all cases identified within the registers with the diagnostic codes X60–X84 or Y10–Y34 (International Classification of Diseases, 10th revision (ICD-10)) for individuals aged 15–24 years in the municipalities of the four counties were included in the study. The ICD-10 codes X60–X84 include attempts or deaths due to intentional self-destructive action, and Y10–Y34 attempts or deaths due to unintentional injury. The individual data linkage was done by the National Board of Health and Welfare.

Additional information about the study population comprising young people aged 15–24years was obtained from Statistics Sweden for each year of the study period (1998–2017). Given the small number of cases – particularly for suicides – in some of the years, we organized the data into five consecutive periods (1998–2001, 2002–2005, 2006–2009, 2010–2013, 2014–2017). To calculate the denominator for each of the five periods, the mean population for each period was obtained and multiplied by four.

### Exposure

A variable representing place of residence at municipal level was created, which included three groups operationalized according to the size of the population: municipalities with a population of <10,000 inhabitants were considered rural (*n* = 24); those with between 10,000 and 50,000 inhabitants were considered semi-rural (*n* = 14); and those with >50,000 inhabitants were considered urban (*n* = 6).

### Analysis

The total number of attempted and completed suicides were summarized and the crude age-specific incidence rates calculated by residence and gender for each study period. To estimate the suicide incidence rates and the incidence rate ratios, along with their 95% confidence intervals according to the type of residence variable, Poisson regression models were applied and the analyses were carried out separately for men and women. To assess differences in trends, a multiplicative interaction analysis between the residence and period variables was also conducted.

The use of data in the present study were reviewed and approved by the ethical committee at the Regional Ethical Review Board in Umeå (2018/136-32).

## Results

From 1 January, 1998 to 31 December, 2017 in northern Sweden, a total number of 5999 (29.40% male) and 294 (72.11% male) youth aged 15–24 years engaged in self-harming episodes or died because of suicide, respectively.

### Suicide attempts

**Figure 1.**
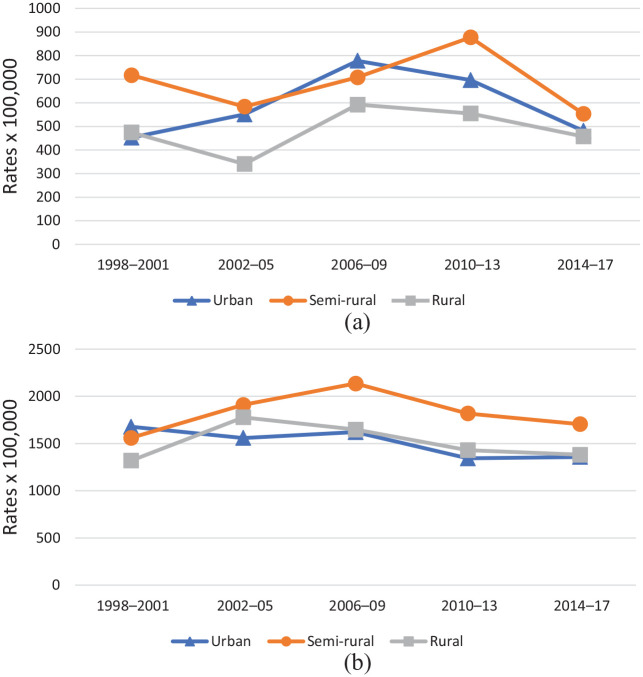
Age-specific crude rates (×100,000) of suicide attempts in men (a) and women (b) by rurality and period, 1998–2017 northern Sweden.

The total number and crude rate of suicide attempts by residence, and stratified according to gender, are presented in Table I and illustrated in Figure 1.

**Table I.** Age-specific crude rates (×100,000) and total number (in brackets) of suicide attempts by rurality, sex and period, 1998–2017 northern Sweden.

	1998–2001	2002–2005	2006–2009	2010–2013	2014–2017
Men					
Urban	452.49 (139)	550.97 (174)	778.05 (263)	696.52 (239)	480.68 (155)
Semi-rural	717.09 (117)	583.65 (97)	708.21 (126)	877.53 (153)	553.05 (90)
Rural	474.71 (40)	340.86 (30)	592.64 (55)	554.83 (49)	457.28 (37)
Total	533.71 (296)	528.06 (301)	729.37 (444)	727.96 (441)	498.14 (282)
Women					
Urban	1677.37 (491)	1558.91 (470)	1620.89 (524)	1342.94 (432)	1358.15 (404)
Semi-rural	1560.18 (223)	1909.92 (282)	2136.56 (337)	1818.24 (279)	1706.28 (236)
Rural	1319.69 (99)	1778.19 (133)	1648.47 (131)	1430.71 (105)	1381.61 (89)
Total	1592.03 (813)	1689.13 (885)	1769.92 (992)	1487.65 (816)	1457.44 (729)

In terms of suicide attempts, the rates were 2–4 times higher among young women compared to young men. Overall, the rates appeared higher in semi-rural municipalities than in urban and rural areas, as well as relatively stable over time, particularly among female youth.

The rates of suicide attempts among young men were highest in semi-rural municipalities in the periods 1998–2001 and 2010–2013, an observation that changed to the disadvantage of urban areas in 2002–2005 (Table II). Among young women, the risk of suicide attempts was higher in semi-rural municipalities for each one of the study periods except the first one (1998–2001), where rural had significantly lower rates than urban municipalities (Table II).

**Table II.** Suicide attempt rate ratios by rurality for each one of the study periods stratified by sex, 1998–2017 northern Sweden.

	1998–2001	2002–2005	2006–2009	2010–2013	2014–2017
Men					
Urban	1.00	1.00	1.00	1.00	1.00
Semi-rural	1.58 (1.24, 2.03)	1.05 (0.83, 1.36)	0.91 (0.74, 1.13)	1.26 (1.03, 1.54)	1.15 (0.89, 1.49)
Rural	1.05 (0.74, 1.49)	0.62 (0.42, 0.91)	0.76 (0.57, 1.02)	0.80 (0.59, 1.08)	0.95 (0.66, 1.36)
Women					
Urban	1.00	1.00	1.00	1.00	1.00
Semi-rural	0.93 (0.79, 1.09)	1.22 (1.05, 1.41)	1.31 (1.15, 1.51)	1.35 (1.16, 1.57)	1.26 (1.07, 1.48)
Rural	0.79 (0.63, 0.98)	1.14 (0.94, 1.38)	1.02 (0.84, 1.23)	1.07 (0.86, 1.32)	1.02 (0.81, 1.28)

In addition, while a significantly downward trend was observed in suicide attempts for young women (but not young men) in urban areas, the pattern appeared stable over time in semi-rural and rural areas, as indicated by the interaction between residence and period.

### Suicide deaths

**Figure 2.**
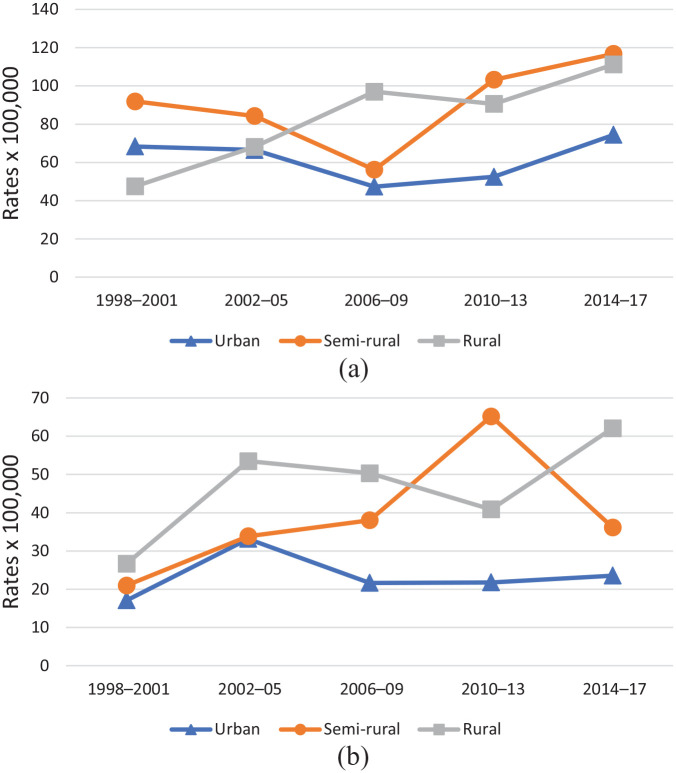
Age-specific crude rates (×100,000) of suicide deaths in men (a) and women (b) by rurality and period, 1998–2017 northern Sweden.

Contrary to suicide attempts, death rates by suicide were higher in men (2–3 times) compared to women. The rates overall were lower in urban municipalities compared to the semi-rural and rural areas, with an increasing tendency over the last two periods, especially in men (Table III and Figure 2).

**Table III.** Age-specific crude rates (×100,000) and total number (in brackets) of suicide deaths by rurality, sex and period, 1998–2017 northern Sweden.

	1998–2001	2002–2005	2006–2009	2010–2013	2014–2017
Men					
Urban	68.36 (21)	66.50 (21)	47.33 (16)	52.46 (18)	74.43 (24)
Semi-rural	91.93 (15)	84.24 (14)	56.21 (10)	103.24 (18)	116.76 (19)
Rural	47.47 (4)	68.17 (6)	96.98 (9)	90.58 (8)	111.23 (9)
Total	72.12 (40)	71.93 (41)	57.50 (35)	72.63 (44)	91.86 (52)
Women					
Urban	17.08 (5)	33.17 (10)	21.65 (7)	21.76 (7)	23.53 (7)
Semi-rural	20.99 (3)	33.86 (5)	38.04 (6)	65.17 (10)	36.15 (5)
Rural	26.66 (2)	53.48 (4)	50.34 (4)	40.88 (3)	62.09 (4)
Total	19.58 (10)	36.26 (19)	30.33 (17)	36.46 (20)	31.99 (16)

In the case of suicide deaths, a significantly higher risk of mortality was observed for both men and women, but only when comparing semi-rural municipalities with urban areas in the period 2010–2013. Although the remaining results were insignificant, higher risks of suicide deaths were found in rural municipalities in young men in 2006–2009, 2010–2013 and 2014–2017, as well as across all periods in young women when compared with urban areas (Table IV).

**Table IV.** Suicide deaths rate ratios by rurality for each one of the study periods stratified by sex, 1998–2017 northern Sweden.

	1998–2001	2002–2005	2006–2009	2010–2013	2014–2017
Men					
Urban	1.00	1.00	1.00	1.00	1.00
Semi-rural	1.34 (0.69, 2.61)	1.27 (0.64, 2.49)	1.19 (0.54, 2.62)	1.97 (1.02, 3.78)	1.57 (0.86, 2.86)
Rural	0.69 (0.24, 2.02)	1.03 (0.41, 2.54)	2.05 (0.91, 4.64)	1.73 (0.75, 3.97)	1.49 (0.69, 3.22)
Women					
Urban	1.00	1.00	1.00	1.00	1.00
Semi-rural	1.23 (0.29, 5.14)	1.02 (0.35, 2.99)	1.76 (0.59, 5.23)	2.99 (1.14, 7.87)	1.54 (0.49, 4.84)
Rural	1.56 (0.30, 8.04)	1.61 (0.51, 5.14)	2.32 (0.68, 7.94)	1.88 (0.49, 7.26)	2.64 (0.77, 9.01)

No significant differences in trends were observed in suicides deaths by place of residence in either gender.

## Discussion

This is the first population-based study in Sweden examining trends of youth suicide attempts and mortality according to gender and municipal place of residence. When compared to the national pattern of attempted suicides in Sweden (113.2 in men and 256.3 in women between 2014 and 2016) [4], the rates in the northern region were higher for the whole period 1998–2017 in young women and men. At the same time, rates of dying by suicides in this study were slightly higher in young men (22.9) but similar in young women (7.9) compared to the national averages for the period 2014–2017 (16.8 and 7.6 for male and female youth, respectively) [3]. Considering the decline of suicide attempts and the fairly stable development of suicide mortality among Swedish youth more generally [3,4], the results from this study reflect a comparable pattern. Specifically, a similarly downward trend was observed in attempts from 2010 onwards for both men and women, and an increase in deaths was found in young men (albeit not among young women) between 2010 and 2017. In relation to the neighbouring Scandinavian countries, the overall rates of youth suicide observed in this study were lower than in Finland but higher than in Norway and Denmark [2].

The overall results from this study correspond to the ‘gender paradox’ in suicidal behaviour rates and the classic international pattern of more suicide attempts among women and more suicide deaths among men [19,20]. To understand these results, Miranda-Mendizabal et al. have identified a number of potentially harmful circumstances in a recent meta-analysis [19]. The authors suggest, for example, that depressive symptoms, eating disorders and exposure to dating violence may partially account for the higher rates of suicide attempts among young women, while drug abuse, externalizing disorders and access to means may explain the higher rates of suicide death among young men. Further investigations are required to establish whether the risk factors outlined in the meta-analysis are more common among youth living in northern Sweden compared to the rest of the country, thereby contributing to the increased rates.

In terms of differences by residential area, the results in this study suggest that semi-rural and rural areas have higher rates of youth suicide deaths compared to urban areas in northern Sweden, especially from the early 2000s onwards. These findings correspond to a body of literature showing a higher incidence of suicide deaths among youth in rural areas [11–14,21,22]. When concerned with attempted suicides, no differences were observed between rural and urban municipalities in this study for either gender. Instead, higher rates were found when comparing semi-rural with urban areas – for the whole period among young women and at specific times among young men. Notwithstanding the scarcity of research on non-fatal suicidal behaviour in youths across geographical areas and despite varying understandings of the definition of rural settings, the results align partly with studies showing non-existent rural–urban gaps in youth suicide attempts or a somewhat lower rate among rural compared to urban youths [13,23,^24^].

Explanations for the higher rates of suicidal behaviours in what were defined as semi-rural municipalities in this study (i.e. those with a population between 10,000 and 50,000) are not clear. In the literature, reduced access to and use of mental health services or a disproportionate burden of suicidal distress in rural areas have been put forward as possible reasons [13]. However, in the Swedish context, these explanations appear to be somewhat inadequate. For instance, it is unlikely that health services would be more difficult to access for youth in semi-rural as compared to rural municipalities, and although suicidal thoughts are a risk factor for attempts [19], no rural–urban differences have been found in stress or suicide ideation for young women or men in northern Sweden [24].

While the data about suicide deaths must be interpreted with some caution due to the low number of cases, the results showing higher risks in rural and semi-rural municipalities, with a worrying increase in the last decade in both men and women, is an issue worthy of further investigation. Specifically, forthcoming studies should identify mechanisms underlying the observed patterns to provide information for targeted and locally appropriate interventions to reduce the gaps.

### Methodological considerations

In the literature, operationalization of ‘rural’ and ‘urban’ geographic areas often differs between studies making comparisons difficult. While a more standardized approach to the categorization of residence might partially resolve this issue, the local contextualization of the concept could be missed and meaningful nuances for policy and practice lost. In addition, although case ascertainment can be a matter of concern owing to the sensitivity of suicide or the lack of enough evidence [25], the Swedish registers are routinely controlled for quality to ensure accuracy and completeness [26]. We expanded the concept of suicide attempts and deaths by including ‘undetermined intent’ (ICD-10 codes Y10–Y34) as part of our case definition. However, it is known that ‘accidents’ (codes V01–X59), ‘homicides’ (codes X85–Y09) and ‘unknown cause’ (codes R95–R99) could also include suicidal outcomes. The extent and nature of measurement bias in the outcomes, particularly in self-harming events, is ultimately unknown. The sudden increase of cases between 2010 and 2013 in the semi-rural areas for both men and women could explain the statistically significant results observed in that period, while the lack of significance in the other period could reflect the low sample size. Despite the Sámi population being a vulnerable group in relation to suicide-related outcomes [27], it was not possible to conduct a sub-analysis in this group given that ethnicity is not officially registered in Sweden. Finally, whether the findings from our study in northern Sweden apply to other parts of the country and/or can be generalized to rural areas in the circumpolar zone remains unclear.

## Conclusions

The results from this study show how attempted and completed suicides tend to be more common in northern Sweden than in the country as a whole. In relation to the overall decline in suicide attempts for both men and women, the higher rate in semi-rural as compared to urban areas requires further investigation. Understanding the increase in suicide mortality among young men and the higher risks for youths in rural and semi-rural municipalities is a public health priority in Sweden that requires close attention. Promoting mental health and well-being jointly with the systematic implementation of suicide prevention programs and locally based monitoring systems – especially in rural and semi-rural areas – may be potential ways forward.
